# Exploring the Relationship Between Psychiatric Illness and Dermatological Disorders: A Narrative Review

**DOI:** 10.7759/cureus.78731

**Published:** 2025-02-08

**Authors:** Alan D Kaye, Rahib K Islam, Abigayle M Castine, Grace C Wester, William T Barham, Catherine G Nguyen, Elena Plakotaris, Bradley Dorius, Julian Kim, Patricia Griffin, Amber Edinoff, Sahar Shekoohi, Giustino Varrassi

**Affiliations:** 1 Anesthesiology, Louisiana State University Health Sciences Center, Shreveport, USA; 2 Medicine, Louisiana State University Health Sciences Center, New Orleans, USA; 3 Medicine, Louisiana State University Health Sciences Center, Shreveport, USA; 4 School of Medicine, Louisiana State University Health Sciences Center, New Orleans, USA; 5 Alcohol, Drugs, and Addiction, Mclean Hospital, Belmont, USA; 6 Psychiatry, Harvard Medical School, Boston, USA; 7 Pain Medicine, Fondazione Paolo Procacci, Rome, ITA

**Keywords:** adhd, biopsychosocial approach, bipolar disorder, comorbidity, dermatological disorders, interdisciplinary care, psychiatric conditions, psychodermatology, schizophrenia, trichotillomania

## Abstract

The interplay between psychiatric conditions and dermatological disorders is complex and multifaceted, often resulting in significant challenges for both diagnosis and treatment. This narrative review examines associations between specific psychiatric conditions, trichotillomania, attention deficit hyperactivity disorder, schizophrenia, bipolar disorder, and various dermatological disorders. The present investigation delves into pathophysiological mechanisms, clinical presentations, and management strategies of these comorbid conditions. Psychiatric disorders can exacerbate dermatological conditions through mechanical trauma, medication side effects, and stress-induced exacerbations, while dermatological disorders can lead to significant psychiatric morbidity. The biopsychosocial approach, emphasizing the importance of integrated care involving dermatologists and mental health professionals, is crucial for improving patient outcomes. This review highlights the need for increased awareness and interdisciplinary collaboration to address the dual burden of psychiatric and dermatological disorders, ultimately enhancing the quality of life for affected individuals.

## Introduction and background

Psychiatric conditions, including trichotillomania, attention deficit hyperactivity disorder (ADHD), schizophrenia, and bipolar disorder (BPD), exhibit significant associations with dermatological disorders [1]. Trichotillomania, characterized by repetitive hair pulling resulting in noticeable hair loss, often coexists with ADHD, as well as obsessive-compulsive disorder (OCD), and manifests with skin-related complications due to the mechanical trauma inflicted during hair-pulling episodes [2]. Similarly, individuals with schizophrenia and BPD frequently experience dermatological issues because of medication side effects, poor self-care, or stress-induced exacerbations of pre-existing skin conditions [3].

Understanding these conditions, epidemiology sheds light on their prevalence and impact. Trichotillomania affects approximately 1%-2% of the population, with onset typically occurring during adolescence [4]. ADHD, a neurodevelopmental disorder, affects up to 5% of children and persists into adulthood in approximately 60% of cases [5]. Schizophrenia and BPD collectively affect millions worldwide, with skin-related manifestations contributing to the burden of these illnesses [6]. The profound impact of psychiatric conditions on quality of life cannot be overstated. Individuals grappling with trichotillomania, ADHD, schizophrenia, or BPD often experience profound emotional distress, social stigma, and impaired functioning due to their symptoms [5,7-9]. Dermatological manifestations further exacerbate these challenges, contributing to feelings of self-consciousness, shame, and diminished self-esteem [10]. Consequently, addressing these disorders' psychiatric and dermatological aspects is paramount to improving patients' overall well-being.

Moreover, the intersection of psychiatric conditions and dermatological disorders poses significant public health challenges. Stigmatization and misconceptions surrounding mental illness and skin conditions persist, leading to barriers to accessing timely and appropriate care [1]. Furthermore, the complex interplay between psychiatric symptoms and dermatological manifestations necessitates a multifaceted approach to treatment, including psychotherapy, pharmacotherapy, dermatological interventions, and psychosocial support [11,12]. Dermatologists play a pivotal role in recognizing and addressing the psychiatric dimensions of dermatological disorders. By adopting a biopsychosocial perspective, dermatologists can provide comprehensive care that addresses the underlying psychiatric conditions contributing to skin-related concerns. Collaboration with mental health professionals facilitates integrated treatment planning, enhancing therapeutic outcomes and mitigating the adverse effects of psychiatric comorbidities on dermatological health [13].

The subsequent sections of this narrative review will explore intricate relationships between specific psychiatric conditions and dermatological disorders, elucidating pathophysiological mechanisms and clinical presentations. Treatment and its implications are beyond the scope of this review.

## Review

Methods

This narrative review was conducted to explore the relationship between psychiatric disorders and dermatological conditions. A comprehensive literature search was performed using PubMed, a widely used biomedical database, to identify relevant studies discussing psychodermatological conditions, their pathophysiology, and the interplay between psychological stress and dermatological manifestations. The literature search was conducted using a combination of Medical Subject Headings (MeSH) terms and keywords related to psychiatric and dermatological disorders. The primary search strategy included terms such as “psychodermatology,” “psychocutaneous disorders,” “psychiatric illness AND skin disease,” “psychophysiologic skin disorders,” “trichotillomania,” “neurogenic excoriation,” “alopecia areata AND psychological stress,” “ADHD AND skin manifestations,” “schizophrenia AND dermatology,” and “bipolar disorder AND dermatologic conditions.” Boolean operators (AND, OR) were applied to refine the search and capture the most relevant studies. The limitation of the study is that, given the narrative review design, this study does not perform a systematic assessment of bias or meta-analysis. 

Pathophysiologic considerations of psychiatric disorders

Trichotillomania

Trichotillomania, defined by the Diagnostic and Statistical Manual of Mental Disorders, Fifth Edition (DSM-5), is a psychiatric condition characterized by repeatedly pulling hair from the scalp, eyebrows, eyelashes, and other sites across the body [2]. The pulling is associated with tension before pulling and relief or gratification following the episode [4]. The episodes may last between a few minutes to a few hours and cause significant distress or impairment of functioning in the patient [7]. When compared to a control group, patients experiencing trichotillomania reported lower self-esteem relating to feeling embarrassed, concerned with appearance, and frustrated [14]. These patients also reported higher levels of distress and lower life satisfaction. The onset of trichotillomania is usually around adolescence, and many report a variety of triggers, including stress, anxiety, boredom, or intrusive thoughts about their hair. Many patients describe that they are typically unaware of their pulling, which is defined as “automatic pulling” [7]. Some patients report that the pulling occurs when seeing or feeling a hair that is described as coarse or rigid. This type of pulling behavior is defined as “focused pulling” [2]. Trichotillomania is commonly misdiagnosed as an anxiety disorder, substance or stimulant use disorder, body dysmorphic behavior disorder, or self-injurious behavior [15]. At the level of the brain, pooled MRI data from peer-reviewed imaging studies has demonstrated that patients with trichotillomania patients had “significant volume reductions of the right amygdala and left putamen and localized morphometric (e.g., curvature) abnormalities of the putamen, caudate, nucleus, and amygdala” [16]. The data indicate a combination of motor control, habit learning, and response suppression implicated via changes to the putamen and directed learning implicated via modifications to the caudate, which all contribute to the pathophysiology of trichotillomania. Additionally, patients with trichotillomania were found to have an abnormally small right amygdala, which is involved in fear processing, arousal, attention, and value representation decision-making. Therefore, negative affective states can trigger or contribute to hair-pulling symptoms [17]. 

Attention-Deficit Hyperactivity Disorder

ADHD is a psychiatric condition diagnosed in children before the age of 12 related to the developmentally inappropriate display of characteristic symptoms, though this can be undiagnosed until adulthood if it is mild. Common symptoms that lead to a diagnosis of ADHD include difficulty concentrating, disorganization, inattention, forgetfulness, and frequent misplacement of items [18]. These symptoms must be present for at least six months and often display a pattern causing dysfunction in multiple settings, such as school, sports, and home. Often, dysfunction caused by ADHD may lead to difficulty with relationships, social integration, emotional regulation, and capacity to make decisions due to significant involvement of the frontal lobe.

From a pathophysiologic consideration, “the anterior cingulate gyrus and dorsolateral prefrontal cortex (DLFPC) are found to be small in cases who are suffering from ADHD,” lending to the lack of driven, goal-directed behavior commonly seen in this population [18]. Other areas of the brain involved include the frontostriatal region, prefrontal cortex, caudate, and cerebellum, all of which appear to have decreased activity on functional MRI (fMRI) in patients suffering from ADHD [5,18]. The prefrontal cortex, part of the frontal lobe, is a significant area of interest for physicians treating patients with ADHD due to its role in executive functioning, planning, organizing, and task management, symptoms that commonly appear to be lacking in a patient with ADHD [19]. Another function of the frontal lobe is inhibiting other parts of the brain that are typically responsible for activity, such as the primary motor cortex and parts of the limbic system. In patients with ADHD, inhibition by the frontal lobe is lost or diminished, leading to the presentation of hyperactive and impulsive symptoms [19]. As a result of diffuse cerebral alterations, both cognitive and functional deficits are present.

Adequate pharmacologic treatment depends on the neurotransmitters dopamine and norepinephrine, which are most frequently implicated in ADHD and are required for maintaining a proper neurochemical environment among the caudate, cerebellum, and prefrontal cortex [5,19]. Primary supporting evidence for this neurochemical theory results from the use of stimulants, such as amphetamines and methylphenidate, to treat common symptoms by increasing physiologic dopamine and norepinephrine concentrations [5,19]. Non-stimulant medications, such as atomoxetine, have also demonstrated symptom improvement by increasing the duration and concentration of norepinephrine in the synapse of multiple brain areas and dopamine concentration in the prefrontal cortex [20]. Atomoxetine treatment has been shown to decrease anxiety and improve personal self-esteem, relationships among family members, and engagement in social situations of children with ADHD [20].

Schizophrenia

Schizophrenia is a disorder characterized by eccentric behavior, psychosis, and social deficits that consists of both positive symptoms, including hallucinations, delusions, thought disturbances, ideas of reference, magical thinking, behavior changes, and perception disturbances, as well as negative symptoms, including anhedonia, lack of motivation, and poverty of speech [8,21]. The etiology and pathophysiology are not well understood due to the complex theory of genetic and environmental risk factor integration that is proposed to evolve into schizophrenia around the second to fourth decades of life [21]. The role of gene contribution is significant, yielding a concordance rate of 40% if both parents have schizophrenia [21]. Similarly, a concordance rate of 46% is present in monozygotic twins, which leaves a significant percentage attributable to non-genetic factors influencing the expression of the disease [8,21].

Like ADHD, an imbalance in neurotransmitters is thought to contribute to the development of schizophrenia. The activities of dopamine, serotonin, gamma-aminobutyric acid (GABA), and glutamate are hypothesized to play a significant role in presenting positive and negative symptoms [21,22]. In this regard, there are four major dopaminergic pathways in the brain, and specific symptoms result if a dopamine imbalance is present. For example, the mesolimbic pathway is hypothesized to produce positive disease symptoms related to “excessive activation of D2 receptors” [21]. The nigrostriatal pathway is theorized to “cause motor symptoms via their effect on the extrapyramidal system” in an environment of low dopamine [21]. Moreover, the mesocortical pathway, under the concentration of lower-than-average dopamine, is theorized to contribute to negative symptoms of the disease [21,22]. Finally, under low dopamine levels, an unregulated tuberoinfundibular pathway may result in high levels of prolactin, leading to amenorrhea in some patients [21]. This can also be a side effect of antipsychotic medications. Another theory for the development of schizophrenia focuses on in-utero alterations in the structure of the brain leading to an absence of cerebral gliosis, which presents with various motor and cognitive differences before diagnosis [21]. In this regard, the last theory is concerned with neuroanatomical changes seen in imaging following a diagnosis of schizophrenia. Cognitive symptoms are a significant component of the disease and could be attributed to frontal lobe abnormalities seen on PET, CT, or fMRI [21,23]. Temporal and parietal lobe atrophy of neuronal cell bodies in the cortical gray matter is also commonly seen [21].

Bipolar Disorder

BPD, like schizophrenia, is theorized to result from the interaction of specific genes with environmental risk factors, yielding a variety of symptoms associated with the spectrum of the disease [24]. Classically referred to as manic depression, BPD is a mood disorder that consists of features from both mania and depression [25]. Mania symptoms include irritability, inability to sleep or think, flight of ideas, euphoria, increased energy, grandiosity, and impulsivity [9]. Depressive symptoms include feelings of hopelessness, lack of interest and pleasure in hobbies, decreased energy, decreased concentration, and inability to sleep. The fluctuation of manic or hypomanic and depressive episodes helps physicians diagnose BPD, a multifactorial disease process developing as a result of both environmental and genetic influences [24].

Several genetic factors have been linked to the pathophysiology of BPD. Non-coding RNA molecules are one example of a genetic risk factor for the development of the disease due to the alterations they cause in epigenetics, resulting in the conformational change of chromatin and RNA editing [24,26]. Epigenetic structural changes result in alternative splicing variants in the RNA genome downstream from the changes in non-coding RNA molecules [24,27]. One study found that among medial frontal gyrus, brain autopsy results from brains from patients without a psychiatric diagnosis and patients with BPD, the expression of non-coding RNA transcripts varied, demonstrating “a global higher number of alternatively spliced variants” present in patients with BPD [24]. Thus, more research is warranted to qualify and quantify the impact of changes in non-coding RNA molecules on BPD development and symptom presentation.

In addition to non-coding RNA molecules, an increase in the dysregulation of microRNA and circular RNA has been observed in the peripheral blood and brain tissue of patients with BPD [24,28,29]. Circular RNA molecules possess significant stability due to their circular structure, which lacks polyadenylation, preventing the action of exoribonucleases [24]. An up regulation in NEBL and EPHA3 genetic loci, two circular RNA molecules, has been noted in patients with BPD [24]. The EPHA3 loci, as well as other genes that produce tyrosine kinase Eph receptors, aid in the neurodevelopment of the CNS via the production of dendritic spines, synapses, and neurotransmitters [24,30]. Eph receptors are associated with anxiety regulation and memory formation. In this regard, an increase in circular RNA coding for these receptors can lead to extremes of anxiety and alterations in memory, both of which are common symptoms in patients who have BPD [29]. Thus, detecting circular RNA molecules may aid in diagnosis [24].

In contrast to the upregulation of the EPHA3 loci, circHomer1a, another circular RNA molecule, is downregulated in the prefrontal cortex of these same patients [24]. CircHomer1a, a known regulatory molecule, is involved in intellectual plasticity and modulating neuronal action at the synapse level [31]. Most notably, circHomer1a influences “neuronal excitability and synaptic plasticity” [24]. As a result of in-vivo circHomer1a knockout, Zimmerman et al. observed an alteration in learning with associated behavioral deficits, thus indicating cognitive dysfunction caused by abnormalities in the identified molecule [32]. Further studies are needed to determine the degree of downregulated circHomer1a in humans, which may result in a display of bipolar symptoms and disease progression.

Psychological stress in association with dermatological conditions

Skin disorders are one of the most common disorders present in society; approximately 85 million Americans have visited a physician for a skin disorder in a year-long period [33]. The pathophysiology of dermatologic diseases is often multifactorial, with effects coming from environmental, genetic, and immunologic sources. Psychologic stress contributes to the risk of dermatological conditions by disturbing the homeostasis of the epidermal permeability barrier [34]. These conditions are broken down into three subgroups: psychophysiological disorders, primary psychiatric disorders, and secondary psychiatric disorders [1].

Psychophysiological Disorders

Psychophysiological disorders are dermatological conditions that can be exacerbated by stress but are not directly caused by any psychiatric condition. Psoriasis is an example of a psychophysiological disorder [1]. Psoriasis is an autoimmune condition characterized by self-reactive T-cells that stimulate an array of cells to proliferate into a thickened epidermis [35]. For example, myeloid dendritic cells are activated and secrete IL-12 and IL-23, promoting the differentiation to TH1 cells and the proliferation of TH17 and TH22 cells [36]. IL-23 signaling plays a role in the transcription of inflammatory mediators through signaling of Tyk2-Jak2 and STAT3 [37]; this leads to skin thickening, increased angiogenesis (causing the reddening of the skin affected by psoriasis), and increased immune cells in the skin [38]. Additionally, keratinocyte proliferation occurs rapidly, and terminal differentiation is incomplete. As a result, these keratinocytes do not release the appropriate amount of extracellular lipids that normally build the connection between differentiated keratinocytes; the stratum corneum is unable to adhere correctly, leading to the classic appearance of flaking skin in psoriasis [35].

Atopic dermatitis (AD) is another example of a psychophysiological disorder; it is associated with increased depressive symptoms and severe stress [39]. The disease is generally more prevalent in children, as it affects 15%-20% of children and 1%-3% of adults in the world, and the prevalence has been increasing. AD presents as flare-ups of cracked, itchy skin [40]. AD is linked to a mutation in the Filaggrin gene; the filaggrin protein functions to align keratin intermediate filaments [41]. A loss of function mutation can result in the ability of allergens to infiltrate the skin, although not all patients with AD have the mutation. There is an upregulation of IL-4 and IL-13, mediated by TH2 cells. These cytokines facilitate the class switching to IgE, promoting the allergy symptoms present in AD [42].

Secondary Psychiatric Disorders

Alopecia areata (AA) is an example of a secondary psychiatric disorder in which a psychiatric disorder may arise from the skin disease. AA is non-scarring hair loss related to autoimmunity, frequently in a small round area on the scalp, which can develop into hair loss throughout the body [43]. Patients generally report onset by 40 years of age [44]. The hair follicle is an area of immune privilege with no lymphatic drainage, downregulation of major histocompatibility complexes, decreased antigen-presenting cells, and natural killer cells [45]. AA is a failure of the immune privilege related to increased MHC-I and MHC-II and resulting attack of the hair follicle by CD8+ T cells, preventing the regeneration of follicles [46]. Stress is a cause of the precipitation of AA by increased corticotropic-releasing hormone receptors, increasing the autoimmune response that is responsible for hair loss [47].

Intersection of psychiatry and dermatology: understanding psychodermatological disorders

Psychodermatology, the study of the association between psychiatric and dermatological disorders, also can categorize conditions by etiology [11]. Psychophysiologic disorders refer to skin disorders responsive to emotional stress, such as urticaria, rosacea, AD, or AA. On the other hand, primary psychiatric disorders are described as isolated delusions involving a skin complaint, such as delusions of parasitosis, neurotic excoriations, trichillomania, or factitical dermatitis. Finally, secondary psychiatric disorders are psychiatric complications experienced as a result of having a skin disorder, such as depression, psychological distress, or social distress.

Previous research into psychocutaneous disorders has investigated the link between the immune system, the hypothalamic pituitary adrenal stress axis, and various neurotransmitters in the dermatological system [48]. AD, one of the most common dermatological conditions in the United States, is a classic example of a psychophysiological disorder exacerbated by increased levels of psychological stress, anxiety, and depression [49,50]. POMC-derived peptides, glucocorticoids, and 11β-hydroxysteroid dehydrogenase activity, mediators of the HPA axis in peripheral tissue, are thought to be the effectors of a maladaptive long-term response to stress that increases Th2-mediated inflammation and compromises stratum corneum barrier function, which predisposes a person to epidermal layer compromise. Paradoxically, these same neuroinflammatory mechanisms may prevent cutaneous dermatoses through the anti-inflammatory activity of endogenous glucocorticoids in the short term [51]. Thus, depending on the chronicity of the stressor, psychological distress can contribute to the precipitation of inflammatory dermatoses.

Conversely, primary psychiatric disorders result in self-inflicted dermatoses, which can be conscious or subconscious. Body-focused repetitive behaviors, dermatitis artifacts, and excessive skin picking may mimic and co-occur with the primary, psychophysiological dermatological disease, further complicating the management and classification of psychocutaneous dermatoses [52]. Though the terminology and categorization of the self-inflicted dermatoses of primary psychiatric disorders is a subject of ongoing debate, joint efforts from psychiatrists and dermatologists in the European Union have aimed to decrease confusion around nonspecific terminology used in the field [53].

Among psychogenic dermatoses, neurogenic excoriation (e.g., skin picking) disorder represents a well-documented condition receiving increased awareness in recent years, being added for the first time to the DSM-5 [54]. Like other cutaneous manifestations of primary psychiatric disease, such as delusions of parasitosis or trichotillomania, treatment is usually directed at the underlying psychiatric disorder, with either behavioral therapy or pharmacologic intervention [11]. Related to psychological assessment not being a routine part of dermatologic clinical practice, multidisciplinary teams involving a psychiatrist and specific dedicated psychodermatological healthcare teams have been proposed as clinical models for addressing psychodermatoses [12]. Additionally, though psychodermatoses are not often characterized as life-threatening, secondary psychiatric disorders stemming from a primary skin disorder, such as depression or anxiety, can result in significant decreases in quality of life or even suicidal ideation, again highlighting the need for multidisciplinary, dedicated psychodermatological health care teams and increased education for dermatologists, psychiatrists, and other health care providers who treat these patients [55]. Finally, there are potentialities for a strong influence of these problems on other aspects largely sensible to psychological alterations, like chronic pain [56].

## Conclusions

The connection between psychiatry and dermatology offers much room for future investigation. The interplay between psychiatric conditions such as trichotillomania, ADHD, comorbid schizophrenia, BPD, and dermatological manifestations is intriguing and provides a strong argument for a multidisciplinary approach to diagnosis and treatments. By synthesizing existing knowledge and fostering interdisciplinary dialogue, clinicians will better understand the complex relationship and the interplay between psychiatric and dermatological disorders, ultimately improving long-term patient outcomes and quality of life.
